# Spontaneous recovery from post‐COVID‐19 brain fog

**DOI:** 10.1002/pcn5.169

**Published:** 2024-01-24

**Authors:** Teruaki Hayashi, Masaaki Iwata

**Affiliations:** ^1^ Department of Neuropsychiatry, Faculty of Medicine Tottori University Yonago Japan

**Keywords:** brain fog, cognitive dysfunction, COVID‐19, follow‐up, post‐acute sequelae of SARS‐CoV‐2

## Abstract

**Background:**

One‐third of individuals who contract novel coronavirus disease 2019 (COVID‐19) reportedly experience persistent symptoms, including respiratory issues, headache, dizziness, taste disorders, fatigue, and various psychiatric and neurological symptoms, known as post‐acute sequelae of SARS‐CoV‐2. In this case report, we present a patient who became aware of brain fog, which is cognitive impairment, approximately 2 months after their COVID‐19 symptoms had resolved, accompanied by anxiety and depression.

**Case Presentation:**

The patient, a 35‐year‐old Japanese man, was infected with COVID‐19 and resumed work approximately 2 weeks later after symptoms improved. Approximately 1 month after returning to work, the patient's concentration became impaired and he started making noticeable errors at work. These symptoms did not improve, leading him to the outpatient clinic specializing in COVID‐19 sequelae at our hospital. Here, he underwent blood tests, electroencephalography, and head magnetic resonance imaging, which did not reveal any abnormalities. Cognitive decline due to COVID‐19 sequelae was therefore suspected, prompting his evaluation in our department approximately 5 months after his initial COVID‐19 infection. Detailed cognitive function tests were performed. He was monitored without the use of medications, and his cognitive function gradually improved. Approximately 11 months after his initial COVID‐19 infection, the same cognitive function tests were conducted again, because his subjective cognitive function symptoms had disappeared, and improvement was observed in many items.

**Conclusion:**

Since brain fog is a relatively common sequela, we emphasize the importance of keeping this in mind from the initial consultations and comparing results over time.

## BACKGROUND

One‐third of individuals who contract novel coronavirus disease 2019 (COVID‐19) reportedly experience persistent symptoms, including respiratory issues, headache, dizziness, taste disorders, fatigue, and various neurological and psychiatric symptoms, known as post‐acute sequelae of SARS‐CoV‐2 (PASC). These symptoms can persist for several months after the infection, regardless of the severity of the initial COVID‐19 illness. This suggests that even patients who did not require treatment during the acute phase may still develop new or long‐term symptoms.^1^

Brain fog, a common manifestation in PACS patients, is primarily a subjective symptom that is not easily understood by others and resists precise cognitive function assessments. In this case report, we present a patient who became aware of cognitive impairment approximately 2 months after their COVID‐19 symptoms had resolved, accompanied by anxiety and depression. With a focus on brain fog from the initial examination, we conducted a comprehensive cognitive function test. We aim to document the course of this case.

## CASE PRESENTATION

The patient, a 35‐year‐old Japanese man with no history of psychiatric illness, contracted COVID‐19 despite having received three doses of a coronavirus vaccine. He initially presented with a fever of 38°C, myalgia, and a headache (described as a feeling of heaviness in the back of the head). He visited a clinic near his home, where he tested positive for COVID‐19. No antiviral medication was administered, the fever subsided within a few days with antipyretic drugs, and the headache gradually improved. Only mild coughing persisted. After approximately 2 weeks, he resumed work as his home quarantine period concluded. Approximately 1 month after returning to work, the patient's concentration became impaired and he started making noticeable errors at work. In addition, he experienced brief episodes of unconsciousness while working on emails and occasionally lost track of his ongoing activities. As a result, he had to take time off work again. These symptoms did not improve, leading him to the outpatient clinic specializing in COVID‐19 sequelae at our hospital. At the time of consultation, there were no physical symptoms such as cough, shortness of breath, or fatigue that could be considered COVID‐19 sequelae. Blood tests did not reveal abnormal findings such as white blood cell counts or C‐reactive protein, and head magnetic resonance imaging showed no abnormal findings suggestive of cognitive impairment, therefore no physical medical treatment was performed and he was referred to a neurologist. There, neurological findings were normal and an electroencephalography was performed, which revealed no abnormalities. Cognitive decline due to COVID‐19 sequelae was suspected, prompting his evaluation in our department approximately 5 months after his initial COVID‐19 infection. At the time of this visit, physical symptoms such as cough and headache, which were thought to be COVID‐19 sequela, had resolved spontaneously. He continued to feel uneasy about being a nuisance at his workplace, which he had felt since the COVID‐19 infection, but it was not a pathological condition. He was able to conduct housework and childcare, and even enjoyed watching movies for short periods of time. Therefore, there was no comorbid anxiety or depression, and we considered this cognitive impairment to be brain fog caused by COVID‐19 infection rather than a psychiatric disorder. Detailed cognitive function tests (Table 1) were performed for this purpose. He was monitored without the use of medications, and his cognitive function gradually improved. After a 3‐month absence from work, he returned to work. Approximately 11 months after his initial COVID‐19 infection, the same cognitive function tests were conducted again, revealing improvements in multiple aspects. At the time of writing this report, approximately 18 months had passed since the initial COVID‐19 infection, and no relapse or recurrence of symptoms had been observed.

**Table 1:** Cognitive function tests.

	First time (5 months after COVID‐19 infection)	Second time (11 months after COVID‐19 infection)
Mini Mental State Examination	29	29
Hasegawa's Dementia Scale‐Revised	27	30
Japanese version of the Montreal Cognitive Assessment	29	29
Frontal assessment battery	18	18
Behavioral assessment of the dysexecutive syndrome	9	1
Noise‐generated CPT		
Non‐response error	0.8%	0.0%
Misinterpretation error	1.0%	0.2%
Multiple response error	1.8%	0.0%
Anticipatory response error	1.4%	0.0%

*Note*. Noise‐generated CPT: CPT software developed to examine the nature of inattention and impulsivity in more detail by modifying conventional CPT and presenting auditory and visual noise simultaneously with standard stimuli. It takes approximately 15 min. The first computer software in Japan to more objectively diagnose inattention and impulsivity, which are considered characteristic symptoms of attention‐deficit/hyperactivity disorder. CPT: Continuous performance pest. A test developed to objectively assess sustained attentional focus. The test consists of pressing a button only for a certain number of presented stimuli.

## DISCUSSION

Individuals infected with COVID‐19 frequently report cognitive dysfunction, often described as brain fog.^2^ Brain fog lacks a precise medical term and is generally described as a “fog‐like state within the head”. It is a general term used to describe a subjective condition in which patients are aware of reduced cognitive function, poor concentration, and lack of mental clarity.^3^, ^4^, ^5^ However, there is no standard definition or diagnostic criteria.^6^

Brain fog can also manifest in other conditions affecting the central nervous system, such as chronic fatigue syndrome and fibromyalgia,^7^ as well as during treatments that impact the immune system, such as cancer chemotherapy.^5^, ^8^, ^9^ Historical references also suggest its presence during the 1918 Spanish flu pandemic^10^ and in cases of severe acute respiratory syndrome coronavirus and Middle East respiratory syndrome coronavirus.^11^, ^12^

While there is no evidence that brain fog is caused by the virus directly infecting the central nervous system, it is known that COVID‐19 can lead to endothelial damage and disruption of the blood–brain barrier.^13^ In addition, inflammation in the choroid plexus can activate microglia in the brain via the complement pathway,^14^ potentially contributing to various neurological disorders, including cognitive dysfunction. These factors are considered as possible causes of various neurological disorders, including cognitive dysfunction.

Predictive models are being developed for patients at risk for cognitive impairment after COVID‐19. It has been reported that elevated fibrinogen and D‐dimer in blood tests are associated with cognitive impairment.^15^ However, in this case, fibrinogen was not measured and D‐dimer was normal.

Cognitive impairment in this case is detailed in Table 1. The Dysexecutive Questionnaire (DEX), part of the Behavioral Assessment of the Dysexecutive Syndrome, is a 20‐item questionnaire designed to identify issues often associated with executive dysfunction. Each item is rated on a 5‐point scale from “not at all (0)” to “almost always (4).”^16^ In this case, the DEX score was 9 out of 100, although it appeared to have improved from 9 to 1, minimal executive dysfunction initially. The noise‐generated continuous performance test (CPT) was slightly low but showed evident improvement, reflecting the improvement of attention impairment caused by COVID‐19. The noise‐generated CPT^17^ was originally developed to assess attention in attention‐deficit/hyperactivity disorder through school age and is not designed to assess attention disorders in adults, as in this case. However, since this was a longitudinal, intra‐individual comparison, not a cross‐sectional one, there were no problems with its use. We are aware, however, that it is not standard practice to use the DEX for executive function assessment or the noise‐generated CPT for attention assessment. At our institution, these were the only tests available to assess attention and executive function, so we had to use them. If a more comprehensive neuropsychological examination could have been performed, it may have been possible to assess thought, cognition, memory, action, and attention in detail. Studies evaluating cognitive impairment post‐COVID‐19 generally report deficits in the cognitive domains of attention and executive function.^18^ In this case, the DEX was low at 9 points even in the extreme phase, so it was difficult to determine whether a decrease to 1 point was sufficient to indicate improvement in executive function. Deficits in attention are consistent with the reported deficits in the early phase, typically within the first month after COVID‐19 diagnosis.^1^ In this case, cognitive testing occurred in the fifth month post‐COVID‐19, but as described previously, approximately 1 month after returning to work the patient's concentration decreased and work‐related errors were noticeable, which can be considered as the time of onset of cognitive impairment.

Cognitive impairment in PASC patients can persist in those who do not require hospitalization and have not experienced COVID‐19‐related events.^19^ Regarding the course of the disease, cognitive improvement is often observed 6 months after COVID‐19 recovery, although differences from the initial values may persist.^20^, ^21^ In this case, spontaneous resolution of symptoms occurred after approximately 6 months of follow‐up. We believe that early and accurate assessments of cognitive function, coupled with feedback to the patient, contributed to his confidence to return to work.

## CONCLUSION

We present a case of brain fog following COVID‐19 remission. While brain fog is a relatively common sequela, it is not always the primary complaint in psychiatric consultations. We emphasize the importance of conducting comprehensive cognitive function tests, keeping brain fog in mind from the initial consultation, and comparing results over time.

## AUTHOR CONTRIBUTIONS

Teruaki Hayashi led the study, conducted the research, and wrote the original draft. Masaaki Iwata helped shape the study and managed the project.

## CONFLICTS OF INTEREST STATEMENT

The authors declare that there are no conflicts of interest related to this study. We have had the following interests over the past 3 years: Masaaki Iwata has received grant funding from the Japan Society for the Promotion of Science, SENSHIN Medical Research Foundation, Japan Agency for Medical Research and Development, Osaka Gas, and speaker's honoraria from Viatris, Sumitomo Pharma, Otsuka, Meiji‐Seika Pharma, Eli Lilly, MSD K.K., Eisai, Pfizer, Janssen Pharmaceutical, Mochida Pharmaceutical, Takeda Pharmaceutical, and Yoshitomiyakuhin.

## ETHICS APPROVAL STATEMENT

This study was conducted according to the principles of the Declaration of Helsinki.

## PATIENT CONSENT STATEMENT

Written informed consent for presentation of his clinical course was given by the patient.

## CLINICAL TRIAL REGISTRATION

N/A.

## Data Availability

N/A.
